# Novel Method for Image-Based Quantified In Situ Transmission Electron Microscope Nanoindentation with High Spatial and Temporal Resolutions

**DOI:** 10.3390/mi14091708

**Published:** 2023-08-31

**Authors:** Jiabao Zhang, Xudong Yang, Zhipeng Li, Jixiang Cai, Jianfei Zhang, Xiaodong Han

**Affiliations:** 1 Beijing Key Lab of Microstructure and Property of Advanced Materials, Beijing University of Technology, Beijing 100124, China; zhangjiabao96@163.com (J.Z.); yangliuge@bjut.edu.cn (X.Y.); lizhipeng1111@yeah.net (Z.L.); jxcai@bjut.edu.cn (J.C.); 2 College of Electronic Information and Control Engineering, Beijing University of Technology, Beijing 100124, China; 3 Bestron (Beijing) Science and Technology, Co., Ltd., Beijing 102600, China

**Keywords:** mechanical quantification, in situ TEM, nanoindentation, focused ion beam, MEMS chip

## Abstract

In situ TEM mechanical stages based on micro-electromechanical systems (MEMS) have developed rapidly over recent decades. However, image-based quantification of MEMS mechanical stages suffers from the trade-off between spatial and temporal resolutions. Here, by taking in situ TEM nanoindentation as an example, we developed a novel method for image-based quantified in situ TEM mechanical tests with both high spatial and temporal resolutions. A reference beam was introduced to the close vicinity of the indenter–sample region. By arranging the indenter, the sample, and the reference beam in a micron-sized area, the indentation depth and load can be directly and dynamically acquired from the relative motion of markers on the three components, while observing the indentation process at a relatively high magnification. No alteration of viewing area is involved throughout the process. Therefore, no deformation events will be missed, and the collection rate of quantification data can be raised significantly.

## 1. Introduction

In situ transmission electron microscope (TEM) mechanical studies have developed rapidly over recent decades. In situ TEM mechanical stages based on micro-electromechanical systems (MEMS) [1] have drawn more and more attention due to their versatile loading modes [2], flexible sample preparation methods [3,4], and high compatibility with TEM functions (e.g., double-tilt) [5,6,7]. MEMS-based in situ TEM mechanical stages have yielded numerous excellent results in recent years [3,8,9,10,11,12,13].

Meanwhile, quantification is essential for in situ TEM mechanical tests. By combining dynamic deformation images with corresponding stress–strain data, in situ TEM mechanical tests can correlate the nano-to-atomic scale structural evolution of a material with its mechanical properties. The widely used commercial quantitative mechanical experiment platform is developed by integrating mechanical sensors on the basis of probe-type in situ in mechanical sample holders. For example, Hysitron developed the PI 95 probe-type in situ mechanical platform consisting of a three-stage probe positioning system. The end of the platform uses a three-dimensional manual adjustment knob to perform millimeter-level rough adjustment of the probe, the middle section uses a three-axis piezoelectric ceramic tube for nanoscale drive, and the front section is equipped with a capacitive sensor [14]. The mechanical sensor enables the platform to achieve mechanical measurements on the order of ~10 nm/10 μN. The probe is installed at the front end of the sensor, which can realize high-precision mechanical loading on the sample. Shan et al. used the PI 95 quantitative mechanical platform to compress Ni nanopillars, measured the strength change in samples during the compression process, and observed that the internal dislocations of the samples gradually disappeared during the mechanical annealing process [15]. A Swedish nanofactory has also developed a probe-type TEM in situ mechanical platform. The platform adopts the actuator of the inertial slider and the three-axis piezoelectric ceramic tube to realize the accurate positioning of the probe. The diamond indenter is fixed at the front end of the holder [16]. Nafari et al. further designed a mechanical sensor for this mechanical platform, and developed a TEM-Nanoindenter mechanical platform that can quantitatively measure the mechanical data of samples [17]. Bufford et al. used the TEM-Nanoindenter to conduct TEM quantitative in situ nanoindentation experiments on Al samples and studied the effects of the interaction between dislocations and incoherent twin boundaries on the mechanical properties of materials. During the experiment, the yield strength of the material was measured to increase with the increase in the compression times. The real-time TEM bright-field image showed that a large number of dislocations nucleated and accumulated at the twin boundary. Continuous application of stress can make the dislocation pass through the twin boundary. It was directly proved that the improvement in material strength is due to the obstruction of dislocations by twin boundaries [18].

The probe-type mechanical platform has the advantages of flexible and controllable movement of the driving probe, complete mechanical sensors, and low signal noise. It is worth noting, however, that the TEM sample holder generally tilts the sample along two orthogonal axes: the direction of the long axis of the sample holder (α-axis), and the direction of the front of the sample holder perpendicular to the electron beam and the long axis (β-axis). The α-axis tilting can be realized by driving the sample holder to rotate around the axis through the goniometer of the transmission electron microscope. The β-axis tilting requires the built-in double-axis tilting mechanism of the sample holder to drive the front part structure to rotate around the β-axis. In the TEM, the part that can be tilted around the β-axis is located in the narrow space between the upper and lower objective lens pole pieces, and the size of the β-axis tilt angle basically depends on the length of this part. The probe-type TEM mechanical sample holder can be tilted about ±30° around the α-axis, but the large three-dimensional drive system distributed along the long axis of the sample holder cannot rotate around the β-axis, which seriously affects the acquisition of the best diffraction conditions. In the in situ experiment, it is difficult to clearly observe the position, shape, and exact movement process of the defect. This type of experimental platform requires the installation of a complex multi-stage drive device at the probe end. The huge volume causes the sample to rotate only along the α-axis of the holder, but it cannot rotate around the β-axis. The loss of the double-axial tilting function directly restricts the high spatial resolution capability of the TEM.

Due to the small size of MEMS devices, the development of a quantitative indentation system based on MEMS can break through the limitations of β-axis tilt. Mechanical quantification in MEMS-based stages is realized in most cases by designing and fabricating deformation/force-sensing beams in the MEMS structure and acquiring deformation/force data (and in turn, strain/stress data, etc.) from monitoring the deflection of the sensing beams during in situ straining.

There are two routes to acquire the deflection of the sensing beams. One is to incorporate a MEMS-based piezoresistive [1] or capacitive sensor [11,19,20,21] in the MEMS mechanical chip and correlate the deflection with the voltage and capacitance collected during the in situ experiment. However, these sensor-based MEMS stages often rely on demanding chip fabrication techniques, intricate assembling procedures, and a complicated data collecting and processing module to ensure the successful and accurate extraction of deflection information. Moreover, the performance of these sensors is often susceptible to environmental variations (electromagnetic interference, vibration, temperature change, etc.), making it difficult to establish a widely applicable and reliable voltage/capacitance-deflection relation.

An alternative method, which has been adopted in many cases, is to read out the deflection of the sensing beams directly from TEM images [6,22,23,24,25,26]. These image-based quantification methods skip altogether the collection of intermediate parameters (i.e., voltage, capacitance), which, on the one hand, avoids complicated fabrication of sensors on MEMS chip and the construction of a data collection and processing module, and on the other hand, eliminates possible errors introduced by environmental disturbance.

However, image-based quantification also faces drawbacks that severely limit its performance. In general, two sets of sensing beams are needed to acquire both deformation and force. Markers are designed on MEMS chips to reflect the deflection of the beams and calculate the deformation of the sample and the load exerted on it [2,27,28]. Unfortunately, global image drift often occurs due to electron beam radiation, temperature variation, etc. In order to eliminate the influence of image drift, a reference marker linked directly to the MEMS frame is needed to reflect the actual deflection of the sensing beam (with respect to the MEMS frame). However, it has proved difficult to introduce the MEMS-frame reference marker close enough to the sample region. As a result, in order to observe the sample reaction and acquire the deflection of the sensing beam, the magnification must be lowered to include all markers in the TEM images, or the viewing area must be altered back and forth between the sample and the markers. The former will lower the spatial resolution of both the TEM images and the mechanical quantification (i.e., a lower displacement and force resolution due to the greater pixel size). The latter will lower the temporal resolution of both the in situ TEM test (missing portions of the deformation process) and the mechanical quantification (i.e., a lower data collection rate due to a lower frame rate of TEM images).

To deal with the above dilemma, taking in situ TEM nanoindentation as an example, we developed a novel method for image-based quantified in situ TEM mechanical tests with both high spatial and temporal resolutions. By virtue of the precise machining and positioning capability of a focused ion-beam-scanning electron microscope (FIB-SEM) system, a reference beam was introduced to the close vicinity of the indenter–sample region. By arranging the indenter, the sample, and the reference beam in a micron-sized area, the indentation depth and force can be directly and dynamically acquired from the relative motion of markers on the three components while observing the indentation process at a relatively high magnification. No alteration in the viewing area is involved throughout the process. Therefore, no deformation events will be missed, and the collection rate of quantification data can be raised significantly.

## 2. Methods

### 2.1. Principle of Image-Based Quantification

**Figure 1:**
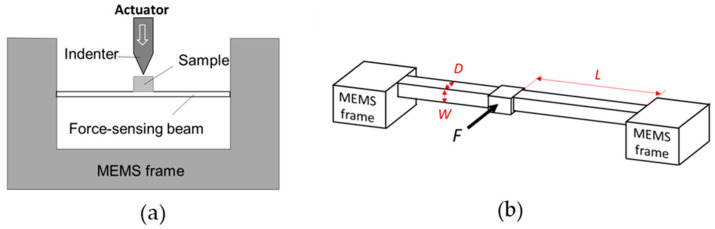
(**a**) Schematic of a quantitative indentation setup; (**b**) schematic of the force-sensing clamped beam.

The abovementioned image-based quantification was first realized on a quantified MEMS chip developed previously by our group [9]. A clamped beam that is often adopted by MEMS mechanical chips [27] was used here. As shown in Figure 1a, the indentation sample was mounted on the center of the force-sensing clamped beam; the indenter was connected to the actuator. When the indenter collides with the sample, the sample transmits the load onto the clamped beam, resulting in deflection of the clamped beam at the center. By measuring the deflection from the TEM image, the load exerted on the sample at the moment the image is acquired can be calculated.

When the indenter moves into the sample, the indentation depth *h* can be calculated by

(1) $$h=y_{I}-y_{s}$$

where *y_I_* is the displacement of the indenter and *y_s_* is the displacement of the clamped beam being pushed by the sample. During the indentation experiment, both *y_I_* and *y_s_* can be accurately obtained from a series of in situ sequential TEM images.

According to the analysis in [1], the load *F* on a clamped beam can be expressed by:

(2) $$F=\frac{2EWD^{3}}{L^{3}}y$$

where *E* is the elastic modulus of the clamped beam; *W*, *D* and *L* are the width, depth, and length of the clamped beam, respectively; and *y* is the deflection of the clamped beam at the center. The structure of the clamped beam and the parameters are shown in Figure 1b. By obtaining *y* at a particular moment during in situ nanoindentation, the load at this moment can be obtained.

### 2.2. Construction of the Image-Based Quantitative Indentation Setup

**Figure 2:**
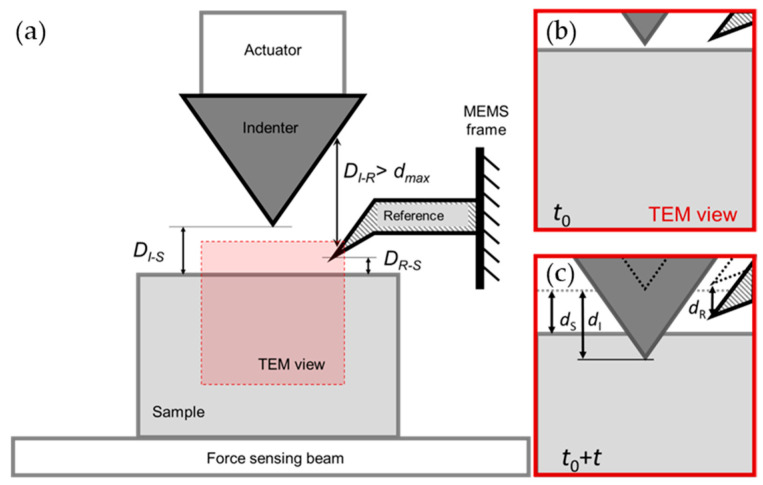
Schematic of the image-based quantitative indentation setup: (**a**) the extracting displacements of the indenter, the sample, and reference from TEM images; (**b**) the relative positions of the indenter, the sample, and the reference beam prior to indentation (*t*_0_) and (**c**) these positions during indentation (*t*_0_ *+ t*).

In order to obtain the actual deflection of the force-sensing clamped beam from TEM images, a reference beam is introduced from the MEMS frame into the close vicinity of the indentation structure as a “fixed point”. The relative displacement of the undeformed region of the sample with respect to the reference beam reflects exactly the deflection of the clamped beam. A schematic of the geometry of the reference beam as well as the relative position of the indenter (*I*), the sample (*S*), and the reference beam (*R*) are shown in Figure 2.

Load and displacement resolutions are critical indicators of the performance of a quantification method. The load and displacement resolutions of image-based quantification depend on the length corresponding to one pixel in TEM images (i.e., pixel size). The pixel size, in turn, depends on both the magnification and the resolution of the TEM images.

Therefore, within an indentation cycle, in order to effectively extract the load-displacement data of the entire process, the reference beam, the indenter, and the observation region of the sample (as indicated by the red square labeled TEM view in Figure 2a) should be in the TEM image at all times. Therefore, the three components should be placed as compactly as possible to allow a higher magnification. Also, the reference beam should be out of the path of the indenter throughout the indentation process with no interference.

Prior to indentation, place the reference beam in the upper-right corner of the TEM image and set to an optimum magnification and ideal imaging condition, as shown in Figure 2b. Define the moment when the indenter touches the sample as *t*_0_ (judging from abrupt variation in sample contrast). Keep the magnification unchanged and do not move the sample via goniometer during the following indentation cycle. Then, the displacements of the indenter, the undeformed region of the sample and the reference beam (denoted, respectively, as *d_I_*, *d_S_*, and *d_R_*) at any *t*_0_ *+ t* relative to their positions at *t*_0_ can be obtained from the in situ images. The displacement of the reference beam is the overall drift of the image. The actuation displacement of the indenter is

(3) $$d=d_{I}-d_{R}$$

The indentation depth is

(4) $$h=\left(d_{I}-d_{R}\right)-\left(d_{S}-d_{R}\right)=d_{I}-d_{S}$$

The deflection of the reference beam is

(5) $$y=d_{S}-d_{R}$$

By substituting Equation (5) into Equation (2), the load detected by the clamped beam is

(6) $$F=\frac{2EWD^{3}}{L^{3}}\left(d_{S}-d_{R}\right)$$

With the above method, the indentation depth *h* and load *F*, i.e., the h-F curve, of the entire indentation process can be acquired from all the in situ TEM images involved, and each data point on this curve corresponds to one TEM image.

## 3. Results and Discussions

### 3.1. Realization of Image-Based Quantitative Indentation

**Figure 3:**
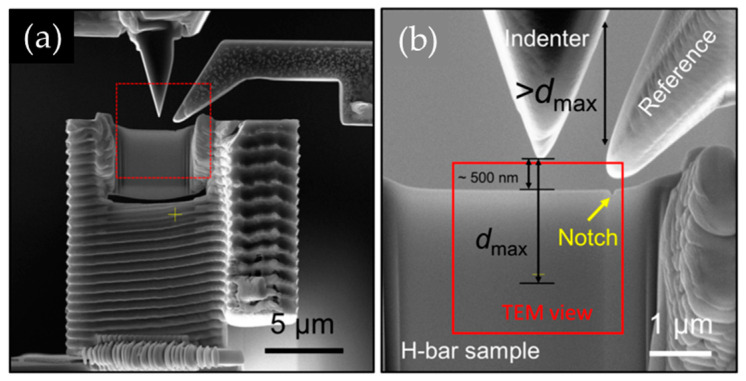
(**a**) FIB preparation of an image-based quantitative indentation structure; (**b**) the enlarged image of the quantitative indentation setup ready for use.

A probe-shaped reference beam was mounted on the MEMS frame and extended all the way to the vicinity of the indenter using FIB (30 kV and 80 pA). Figure 3a is the SEM image of the image-based quantitative indentation setup. As shown by Figure 3b (the enlarged image of the area in Figure 3a marked by the red dashed frame), the reference beam fixated to the MEMS frame extends all the way to the indenter–sample region. To reduce the implantation of Ga ions, an H-bar indentation sample was fabricated in three steps. First, the ion beam was set at 30 kV, ~80 pA, during the sample cutting and transfer process, which enabled the maintenance of a high initial preparation process for the sample. The sample thickness was set to 1500 nm under this condition. Second, before further thinning of the sample, to minimize the implantation of Ga ions, an approximately 1 μm thick protective Pt coating layer was deposited on the sample at 30 kV, ~0.23 nA. Finally, the sample was sliced to the size of ~150 nm at 30 kV, ~40 pA, and the sample surface was cleaned using the mild milling conditions of 15 pA/5 kV and 9 pA/2 kV. The H-bar indentation sample was fixated on the center of the force-sensing clamped beam right under the indenter (30 kV and 40 pA). A small notch was machined using a low-energy ion beam (5 kV and 15 pA) at the upper-right corner of the thinned region of the sample to serve as the marker. The area marked by the red frame is the observing area in the following in situ TEM nanoindentation experiment.

### 3.2. Image-Based Quantitative Indentation Experiment

After a MEMS chip prepared with image-based quantitative indentation setup was inserted into the TEM column, the image-based quantitative indentation experiment was carried out in the following steps:

1. Tilt the H-bar sample to a suitable viewing condition, and make sure the reference beam, the area to be indented and the area of interest on the sample are within the sight of the camera;
2. Set to the highest magnification that meets the above requirements and a suitable image resolution;
3. Set the frame rate;
4. Start recording;
5. After all these preparations, drive the indenter towards the sample. Do not tilt or move the sample with the goniometer during the entire process of indentation, holding, and retraction. Monitor the process to ensure that the indenter, the reference beam, and the observing region of the sample are in the TEM images at all times;
6. After the indentation process, stop recording.

If another cycle of indentation is needed, repeat the above operations. A sequence of TEM images of identical sample tilt and magnification can be acquired for each indentation cycle.

### 3.3. Extraction of Displacement Information

The software used for tracking features in images and extracting displacement information was the image processing software Fiji-ImageJ (fiji-win64) based on Java. Fiji can output the relative displacements (in pixels) of a characteristic region shared by a set of images. Using this function, characteristic regions were selected on the indenter, the reference beam, and the sample, respectively, to extract the trace (in pixels) of the three components during the same indentation process. The traces were then translated from pixels into actual length in nm. The time-indentation depth-load relation of the indentation process can then be obtained by calculation.

In the following text, by taking an indentation cycle of a single-crystalline Ni sample as an example, the data acquisition and processing procedure of the image-based quantification was explained in detail. The TEM observations were performed on a transmission electron microscope (TEM, FEI-ETEM) at 300 kV. The in situ deformation of the sample was performed using an in situ mechanical TEM sample holder (Bestron-INSTEMS-M, Bestron (Beijing) Science and Technology, Co., Ltd., Beijing, China). The in situ deformation was driven by a lead zirconate titanate (PZT) actuator under displacement control. The PZT actuator could provide a full displacement range of 4 μm and a loading rate of 1–2 nm/s.

**Figure 4:**
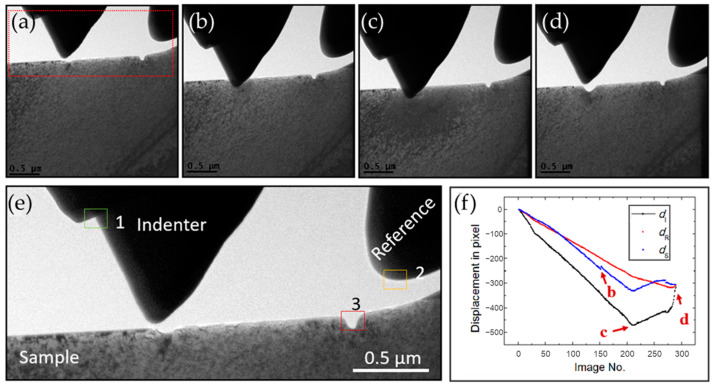
Extraction of the displacements of the indenter, the reference, and the sample in pixels. (**a**–**d**) Sequential TEM images prior to indentation, during indentation, at the greatest indentation depth, and after indentation, respectively; (**e**) a partial view of the red framed area in (**a**), selection of characteristic regions (1–3) on the indenter, the sample, and the reference beam; (**f**) displacement vs. image number of the 3 regions on the indenter, the reference beam, and the sample, respectively.

Figure 4 shows the extraction of the displacements of the indenter, the reference, and the sample from 289 sequential TEM images of the indentation process of the nanoindentation sample shown in Figure 3. Figure 4a–d are the images of the sample prior to indentation, during indentation, at the greatest indentation depth, and after indentation, respectively. The selection of the characteristic region (as a marker) will affect the accuracy of displacement data. As shown in Figure 4e, three characteristic regions were selected on the indenter, the reference beam, and the sample, respectively, to track the trace of the three components throughout all the 289 images involved in this process. Note that the selected regions should appear in all the images. Figure 4f shows the displacement vs. the image number of the three regions on the indenter, the reference beam, and the sample, respectively.

### 3.4. Calculation of Quantitative Mechanical Data

The data in pixels from regions 1, 2, and 3 were transformed into actual length, and the image number was transformed into the actual acquisition time of the image. Note that if the sample was tilted prior to indentation, the projection of the distances measured from TEM images should be adjusted to the actual value. For example, in this case, if the sample was tilted along the beta axis, the displacement in the image is actually the projection of actual displacement and should be adjusted by multiplying by a coefficient of 1/cos *β*.

The pixel size *d*′ (nm/pixel) can be measured from the image directly. Therefore, the actual displacement of the indenter, the reference beam, and the sample are

(7) $$\left\{\begin{array}{l} d_{I}=n_{I}\cdot d^{\prime }/\cos\beta \\ d_{R}=n_{R}\cdot d^{\prime }/\cos\beta \\ d_{S}=n_{S}\cdot d^{\prime }/\cos\beta \end{array}\right.$$

The indentation depth is

(8) $$h=d_{I}-d_{S}=\left(n_{I}-n_{S}\right)\cdot d^{\prime }/\cos\beta$$

The load on the clamped beam is

(9) $$\begin{array}{c} F=\frac{2EWD^{3}}{L^{3}}\left(d_{S}-d_{R}\right)\\ =\frac{2EWD^{3}}{L^{3}}\cdot \left(n_{S}-n_{R}\right)\cdot d^{\prime }/\cos\beta \end{array}$$

**Figure 5:**
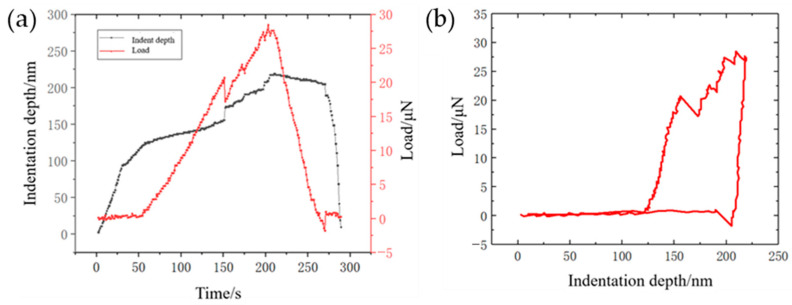
(**a**) Variation curves of indentation depth and load with time; (**b**) indentation–depth–load curve.

In this case, the pixel size *d*′ is 1.4 nm/pixel, and the beta tilt angle is 0. The dimensions of the clamped beam are *W* = 60 μm, *D* = 21 μm, and *L* = 900 μm. The clamped beam is made of single-crystalline silicon, and the elastic modulus along the clamped direction ([110]_Si_) is *E* = 169 MPa [1]. By substituting these parameters into Equations (8) and (9), the time-indentation depth-load relation of the indentation process can be obtained. Figure 5a shows the variation in indentation depth and load with time. Figure 5b shows the corresponding indentation depth–load curve of this process. The displacement resolution, which corresponds to the pixel size, in this case is 1.4 nm, and the load resolution is 0.36 μN. The frame rate of the indentation process is approximately 5 frames/second. Therefore, the image-based quantification method introduced and tested in this article can simultaneously provide relatively high spatial (displacement and load) resolution and temporal (frame rate) resolution.

Since the image-based quantification method is free from any data acquisition and processing module, it can be well applied to other in situ TEM mechanical stages, such as bimetallic strips [29,30,31], thermal actuators [5,28,32], comb drive actuators [6,26], probe-type mechanical holders [33,34,35], etc. All that is required is to mount the sample on a clamped beam and introduce a reference marker to the close vicinity of the sample from a well-chosen stationary location with respect to the fixed ends of the clamped beam, so as to accurately reflect the deflection of the beam. However, since the relevant markers are required to be in the viewing area throughout in situ straining, the magnification cannot be increased much further, making it very difficult to realize atomic-scale quantified straining in TEM. On the other hand, thanks to the rapid development of fast cameras (Themofisher Ceta, Gatan Oneview, Gatan K2/K3, etc.) for TEM, the temporal resolution of this method can be raised further. In addition, automation of this method can be pursued in interdisciplinary research and development with information technology to provide real-time deformation/force data.

## 4. Conclusions

By taking in situ TEM nanoindentation as an example, we developed a novel method for image-based quantified in situ TEM mechanical tests with both high spatial and temporal resolutions. By virtue of the precise machining and positioning capability of the FIB-SEM system, a reference beam was introduced to the close vicinity of the indenter–sample region. By arranging the indenter, the sample, and the reference beam in a micron-sized area, the indentation depth and deflection of the sensing beam can be directly and dynamically acquired from the relative motion of markers on the three components while observing the indentation process at a relatively high magnification. No alteration of viewing area is involved throughout the process; therefore, no deformation events will be missed and the collection rate of quantification data can be raised significantly.
